# Predicting the Effect of the Loading Rate on the Fracture Toughness of Hydraulic Asphalt Concrete Based on the Weibull Distribution

**DOI:** 10.3390/ma17040803

**Published:** 2024-02-07

**Authors:** Jianxin He, Xinyu Ding, Wu Yang, Haihua Yang, Liang Liu

**Affiliations:** 1 Faculty of Water Resources and Civil Engineering, Xinjiang Agricultural University, 311 East Nongda Rood, Urumqi 830052, China; 13677552079@163.com (X.D.); slyangwu@xjau.edu.cn (W.Y.); yanghaihua@xjau.edu.cn (H.Y.); liuliang2023@xjau.edu.cn (L.L.); 2 Xinjiang Key Laboratory of Hydraulic Eng Security and Water Disaster Prevention, 311 East Nongda Rood, Urumqi 830052, China

**Keywords:** hydraulic asphalt concrete, SCB test, *K_IC_*, bending tensile strain, Weibull distribution, loading rate, log-normal distribution

## Abstract

The cracking problem of asphalt concrete panels is a crucial consideration in the design of hydraulic asphalt concrete seepage control bodies. Panels experiencing uneven rises or falls of water levels during impoundment may exhibit loading rate effects. Investigating the fracture toughness value of asphalt concrete under varying loading rates is essential. This study employs a statistical method to calculate the fracture index *K_IC_*, using the semi-circular bending test (SCB) to examine the effect of loading rates on the Type I fracture mode of hydraulic asphalt concrete. The data are analyzed using the two-parameter Weibull distribution curve, offering insights into the minimum number of *K_IC_* test specimens. The results indicate an increase in *K_IC_* with loading rate, with greater data dispersion at faster rates. The Weibull distribution curve successfully fits the fracture behavior under different loading rates, providing valuable predictions. This study estimates the minimum number of SCB test specimens to be nine, based on a confidence level of 0.95 and a relative deviation not exceeding 5%.

## 1. Introduction

Asphalt concrete, known for its good impermeability, is suitable for various water conservancy impermeability projects [1]. During the normal operation of pumped storage power stations, reservoir water levels experience periodic fluctuations [2], impacting asphalt panels. Such a fluctuation causes a certain load change, leading to strength attenuation and cracks in asphalt concrete. Laboratory research on loading rates can simulate field conditions and analyze the crack problem of asphalt concrete with fracture mechanic theory, which can better understand the fracture behavior of asphalt concrete. Therefore, at the design stage, a method that can accurately predict the fracture toughness of asphalt concrete cracks under different loading rates is investigated, so as to achieve the purpose of reducing the number of tests, improving the mix design and effectively predicting the performance of asphalt concrete.

At present, scholars at home and abroad have proposed to use the semicircular bending test (SCB) to evaluate the fracture characteristics of asphalt mixture. The method is based on fracture mechanic theory. It was first proposed by Chong [3], and then Krans [4] applied the SCB test to asphalt mixture. A large number of results show that the fracture index obtained in this test can indicate the crack resistance of asphalt concrete, and it has been unanimously recognized by the academic community [5,6]. Compared with the trabecular bending test, not only is the specimen preparation simple and has strong applicability, but also the permanent deformation of the contact point between the semicircular specimen and the support is smaller [7], which reduces the influence of external causes on the test results. With the deepening of the research, Fu Xin et al. [8] clarified the influence of the mechanical properties of SCB specimens on the test parameters such as thickness, spacing of supports, width and depth of incision, etc., and obtained the appropriate dimensional parameters through numerical simulation; Xiong Aiming [9], Feng Decheng [10], and Jiang Xinlong [6] et al. calculated a variety of fracture indexes based on SCB test results to evaluate the low-temperature cracking resistance of asphalt mixture, and compared the sensitivity of each fracture index under different test conditions; Zhu Hongzhou et al. [11] pointed out that temperature and time can improve the self-healing ability of asphalt mixture using the fracture-healing-fracture test on SCB specimens, but the higher the temperature and the longer the time, the better the healing ability; N. Shahryari et al. [12] compared the effect of three specimen geometries, SCB, Edge Notched Disc Bending (ENDB), and Edge Notched Disc Compression (ENDC), on the Type I fracture behavior of modified and unmodified mixes; other scholars [13,14] have investigated the cracking characteristics of asphalt mixtures with different aging methods using the SCB test. On the other hand, hydraulic asphalt concrete belongs to viscoelastic–plastic materials, and the loading rate becomes sensitive to its comprehensive physico-mechanical properties. Many scholars have investigated the effect of the loading rate on the law of the Type I fracture mode of asphalt mixtures as well as the use of microstructural models to predict the fracture behavior of mixtures [15,16,17,18]. However, most researchers have determined the fracture toughness of asphalt concrete at a given condition from the mean value of a finite number of tests, whereas the non-uniform distribution of coarse and fine aggregate particle sizes between specimens, as well as the uncertainty in the amount of coarse aggregate at the leading edge of the specimen’s pre-cracks, make the test results of this non-homogeneous composite material far more discrete than those of homogeneous materials; only by repeating the test several times will the average of the calculated fracture toughness values be more reliable. Discrete results of fracture toughness values are usually analyzed using probabilistic failure principles, and the Weibull model, a typical probability function for brittle failure [19], is a probability distribution function proposed by the Swedish scientist W. Weibull as an effective model for the discrete analysis of fracture toughness data for asphalt concrete [20]. Scholars have investigated the applicability of Weibull distribution curves in predicting the fracture toughness of different materials such as asphalt concrete [21,22], rock [23,24], polymers [25,26], composites [27], ceramics [28], and carbon fibers [29]. The results show that the Weibull statistical method can provide a better engineering evaluation of the cracking resistance of the above materials.

Therefore, this paper adopts the statistical method, based on the fracture mechanic theory, to carry out the fracture toughness test of hydraulic asphalt concrete under different loading rates, analyzes and predicts the distribution characteristics of asphalt concrete Type I fracture strength under different loading rates using the Weibull model, verifies the reasonableness of the prediction curves, and finally provides the number of and the way to calculate the minimum number of specimens for *K_IC_* in the SCB test, which can provide insights into the fracture behavior of asphalt concrete.

## 2. Test Materials and Methods

### 2.1. Raw Materials and Mixing Ratios

The test utilized a project selected to deliver a lithology of medium-thick layered/thick laminated gray/grayish-white fine-crystalline limestone. Coarse and fine aggregates were produced by crushing the rock using a jaw crusher, and part of the filler was prepared individually using a disc crusher. The asphalt used was 70# road petroleum asphalt produced by “PetroChina Karamay Petrochemical Co., Ltd. (Karamay, Xinjiang Uygur Autonomous Region, China)”, and the various technical properties of the asphalt were tested, and the test results are shown in Table 1; the particle size grading of the mineral grading and the dosage are shown in Table 2.

### 2.2. Specimen Preparation and Test Program

**Figure 1:**
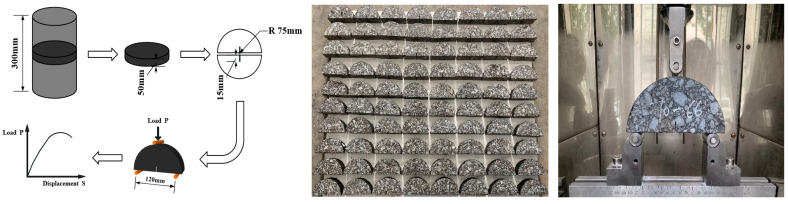
Schematic of the forming and testing processes of SCB test specimens.

The mechanical properties of the SCB specimens tended to be stabilized when their thickness was 50 mm [6,8,30], and in order to reduce the shear deformation at the support point, the support spacing was 0.8 times the specimen diameter [7,8,30]. Based on the above research results, the size of the specimens selected for this study was 150 mm in diameter and 50 mm thick. Specimens were prepared using the compaction method of molding Φ150 × 300 mm specimens, divided into three layers of compaction, and the cuts were then made with a cutter, removing 15 mm at each end to reduce the effect of uneven density, and a pre-crack with a depth of 15 mm and a kerf width of 1.8 mm was cut into the center of the bottom. In order to consider the effect of the loading rate, the prepared specimens were divided into four groups of 20, placed in a room at a constant temperature of 10 °C for more than 48 h, and the universal testing machine was used to conduct the test, and the loading process ensured that the spacing of the pivot point at the bottom of the specimen was 120 mm, and the test temperature was strictly controlled, and the test groups were loaded at the conditions of a 0.2, 1, 5, and 10 mm/min loading rate. A data acquisition system was used to control the whole test via computer and record the load displacement data during the loading process. The specimen molding and test processes are shown in Figure 1.

### 2.3. Fracture Index Calculation

#### Stress Intensity Factor

For the stress intensity factor (*K_IC_*) calculation, fracture mechanic testing focused on the crack expansion of pre-cracked specimens to characterize the crack extension capacity of a material in the presence of structural defects [5]. The critical stress intensity factor was calculated as shown in Equation (1):

(1) $$K_{IC}=\frac{P}{2rt}\times \sqrt{\pi a}\times Y_{I(0.8)}$$

where *K_IC_* is the critical value of the stress intensity factor, MPa·m^0.5^; *P* is the critical load, kN; *r* is the radius of the specimen, m; *t* is the thickness of the specimen, m; *a* is the length of the cutout, m; and *Y_I_*_(0.8)_ is the Type I fracture stress intensity factor.

According to the results of a previous study by Lim [31], *Y_I_*_(0.8)_ can be calculated using the following equation:

(2) $$Y_{I(0.8)}=4.782-1.219(\frac{a}{r})+0.063e^{7.045\frac{a}{r}}$$

## 3. Analysis of Test Results

**Figure 2:**
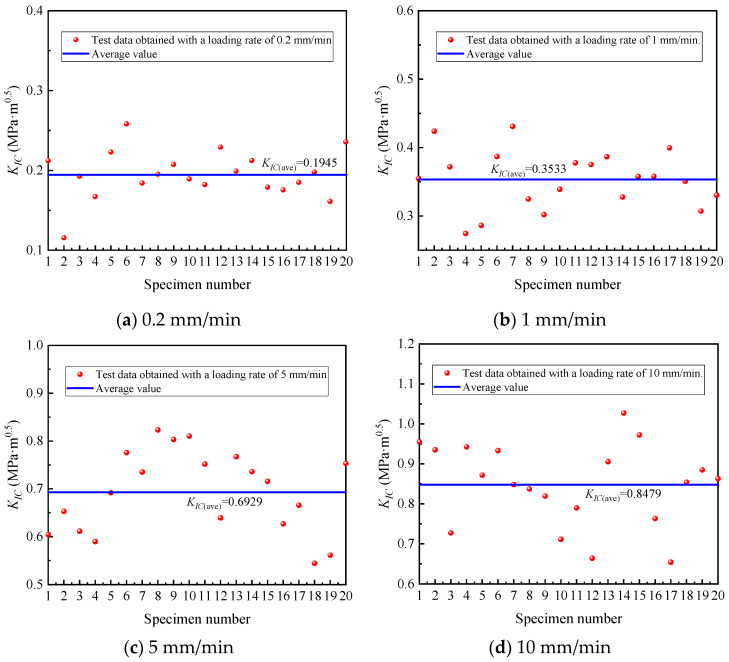
(**a**–**d**) Changes in the *K_IC_* value of SCB specimens under loading rates of 0.2, 1, 5, and 10 mm/min and the corresponding average values of each group.

The *K_IC_* values of hydraulic asphalt concrete at different loading rates are shown in Figure 2, which shows that for any loading rate, the dispersion of each set of results is relatively large due to the non-uniformity of the aggregate inside the asphalt concrete. The variance values of the 20 test results at different loading rates were calculated using the ANOVA method, which was used to indicate the strength of the dispersion of the test results, as shown in Table 3. The degree of dispersion of the *K_IC_* test data increases with an increase in the loading rate, and the degree of dispersion of the data with a loading rate of 10 mm/min has a nearly 10-fold difference in the variance value compared with that of 0.2 mm/min, and showed an increase in strength with the increase in loading rate, which is consistent with the findings in the literature [16]. The spread (the spread between the maximum and minimum values) in the test data is also gradually increasing, which can be seen from the following fact: the larger the rate, the larger the error range. The average *K_IC_* value also increases with it, respectively, 0.1945, 0.3533, 0.6929, and 0.8479 Mpa·m^0.5^. The median is approximately equal to the mean, which means that the loading rate has a significant effect on the fracture toughness value of hydraulic asphalt concrete. The increase in *K_IC_* with the loading rate has also been reported in previous studies on the crack resistance of asphalt concrete materials [7,16,17] with good agreement.

**Figure 3:**
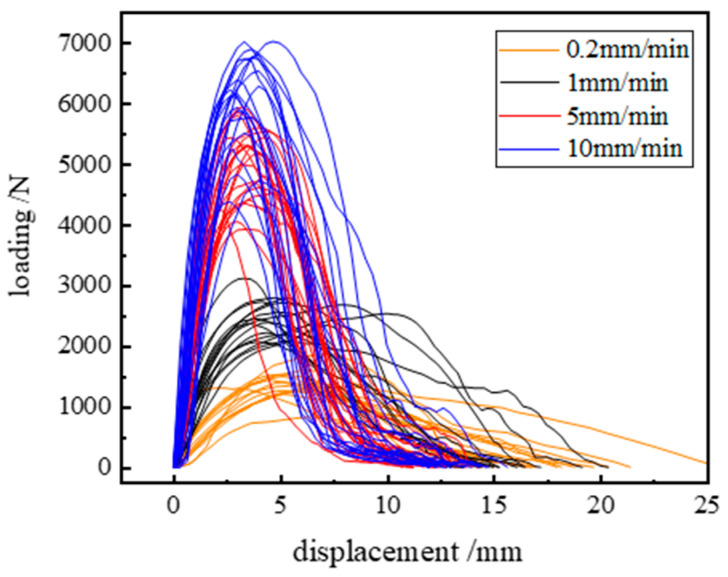
Test load–displacement diagram for different loading rates.

The load–displacement curves of the specimens under different loading rates are given in Figure 3. It can be seen that the fracture behavior and fracture load of asphalt concrete depend on the loading rate: the larger the rate, the stronger the bearing capacity of asphalt concrete. Before the peak load, the damage behavior of the specimen is mainly manifested as a linear elastic fracture, and with the increase in loading rate, the slope of the initial section of the curve is gradually steeper and the area enclosed by the curve is larger, and overall, there is a recurrence of the relationship between the increases. The reason why the curves are not smooth or stable is due to the creation of voids or defects within the specimen during fracture, the beginning of cracks in the holes made to create seams, the interconnection between the seams, or due to the presence of stronger coarse aggregates at the leading edge of the pre-cracks. The large fluctuations between the curves at the same loading rate reflect the large dispersion of the test results, so the shape and trend of the curves can also be used to determine the magnitude of the dispersion of the test results.

### 3.1. Optimal Span

In this section, the applicability of Weibull’s statistical approach to hydraulic asphalt concrete will be examined. The *K_IC_* data obtained from the SCB specimens under four loading rates were classified, and the dispersion of the fracture toughness results was analyzed using the probabilistic failure analysis method, and then the rank sum cumulative probability method was used to obtain the probability of failure *P_f_* of the SCB specimens under different loading rates, and the probability of the failure is denoted as follows:

(3) $$P_{f}=\frac{j-0.5}{N}$$

where *j* is the number of tests sorted in descending order of the fracture toughness value (*j* = 1, 2, 3, …, *N*), and *N* is the total number of tests at each loading rate.

Weibull [32] first proposed a stochastic approach to represent the strength distribution of brittle materials for which damage is mainly caused by the creation and extension of discontinuities, defects, and cracks, describing a probabilistic behavior of fractures that follows the weakest link theory. The two-parameter model of the Weibull distribution for fracture toughness is usually written as follows:

(4) $$P_{f}(K_{IC})=1-EXP\left[-{\left(\frac{K_{IC}}{K_{0}}\right)}^{m}\right]$$

where *K_IC_* is the critical value of the critical stress intensity factor (or fracture toughness value), MPa·m^0.5^; *K*_0_ is the normalization factor, which is equal to the value of *K_I_*, and *K_I_* indicates that the probability of failure is 0.623; and m is the fitting parameter describing the magnitude of the deviatoricity.

Two unknowns need to be calculated in the two-parameter Weibull failure probability distribution to establish the relationship between the failure probability and the fracture toughness value, which need to be taken twice logarithmically by rearranging the terms in Equation (4), and the expression becomes the following:

(5) $$\ln\ln\left(\frac{1}{1-P_{f}(K_{IC})}\right)=m\ln(K_{IC})-m\ln(K_{0})$$

**Figure 4:**
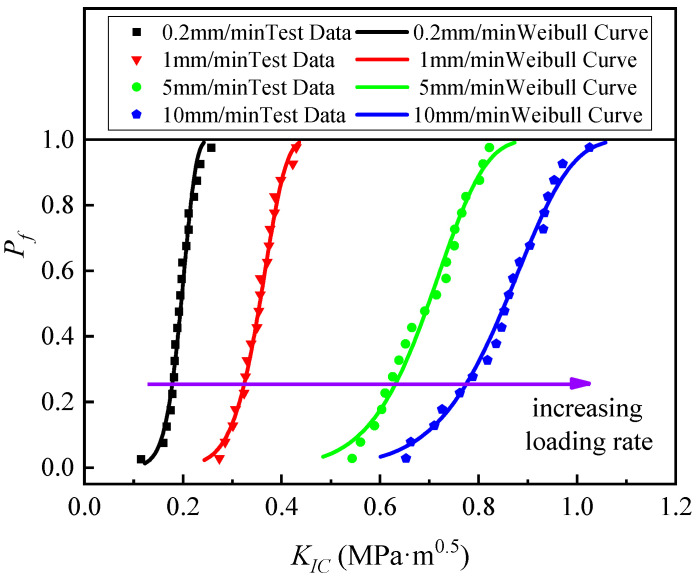
Weibull distribution curve of asphalt concrete test results under different loading rates.

In fitting the test data using the least squares method using Equation (5), the Weibull model parameters were determined for each group of SCB specimens at loading rates of 0.2, 1, 5, and 10 mm/min and summarized in Table 4. The *K_0_* values of the SCB specimens at a loading rate of 10 mm/min are greater than the Weibull parameters at lower loading rates. The fracture toughness results from the tests were plotted as *P_f_ − K_IC_* plots and fitted with the calculated Weibull distribution curves (shown in Figure 4). A good Weibull distribution curve can be obtained to express the fracture behavior of asphalt concrete using 20 test specimens. By comparing the test data under different loading rates, it can be seen that for each set of fracture toughness data, special Weibull curves with specific Weibull parameters were obtained, and the different curves reflect the effect of different loading rates on the fracture-resistant properties of hydraulic asphalt concrete. It can also be seen from the figure that as the loading rate increases, the Weibull distribution curve moves to the right. Using the change rule on the Weibull curve, it was found that with loading rates of 5 and 10 mm/min, the asphalt concrete fracture toughness upper limit and lower limit values of the change range are larger, that is, the curve is more gentle, which is further proof of the following conclusion: the greater the loading rate, the greater the discretization. In summary, the two-parameter Weibull probability curve fits the hydraulic asphalt concrete fracture toughness values well and with high accuracy.

**Figure 5:**
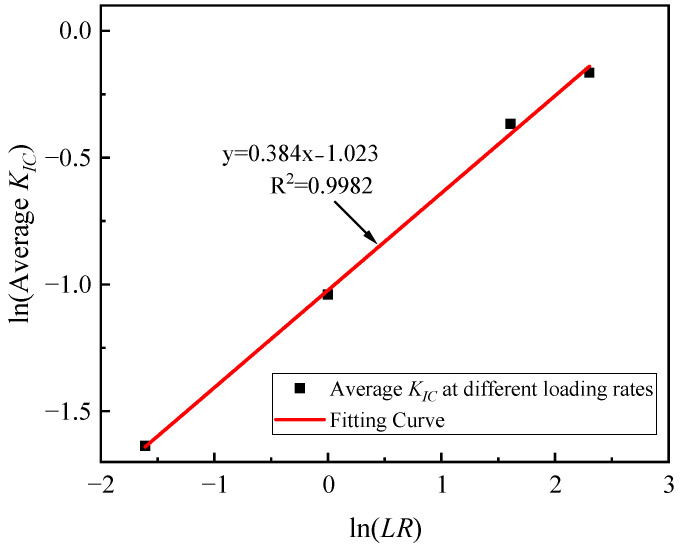
Relation between the loading rate of asphalt concrete and average fracture toughness value.

Based on the fracture toughness tests performed, it can be observed that the average *K_IC_* value is directly proportional to the loading rate, and further analysis reveals that the average fracture toughness value shows a simple linear relationship with the logarithm of the corresponding loading rate, as shown in Figure 5, and based on this empirical relationship, the average *K_IC_* value of asphalt concrete can be written as a function of the loading rate (see Equation (6)):

(6) $$K_{IC(\mathrm{ave})}=e^{0.384Ln(LR)-1}$$

where *LR* is the loading rate, mm/min. The displacement factor is defined as follows:

(7) $$S.F=\mathrm{Shift}\;\mathrm{factor}=\frac{K_{IC(\mathrm{ave})}}{K_{IC\left(\mathrm{ave}\right)\left(LR=1\mathrm{mm}/\min\right)}}$$

Given a desired *LR*, the average *K_IC_* can be determined using Equation (6), and based on Equation (7), a simple displacement is then determined, and *LR* = 1 mm/min is specified as the reference data. The Weibull parameter of the asphalt concrete tested at the desired loading rate (*K*_0_) can be determined from the following equation based on the Weibull parameter of the reference case:

(8) $$\frac{K_{IC\left(\mathrm{ave}\right)\left(LR=\mathrm{Expectation}\right)}}{K_{IC\left(\mathrm{ave}\right)\left(LR=1\right)}}=\frac{K_{0(LR=\mathrm{Expectation})}}{K_{0(LR=1)}}=S.F$$

### 3.2. Rationalization Verification

In order to verify that hydraulic asphalt concrete can predict the Weibull distribution curve under different loading rates through the displacement factor, two groups of SCB tests with different loading rates (0.05 and 3 mm/min) were conducted again in this study: 20 tests in each group, and *LR* = 1 mm/min was specified as the reference data. Equations (6) and (7) were used to predict the two-parameter Weibull curves of SCB specimens at *LR* = 0.05 and 3 mm/min, and from Equation (6), the average *K_IC_* was calculated to be 0.116 and 0.561 MPa·m^0.5^, respectively, and Equation (7) determined that the displacement factors, S.F, were determined to be 0.330 and 1.588, respectively, and thus the calculated values of *K*_0_ are as follows:

$$K_{0(LR=0.05)}=S.F\times K_{0(LR=1)}=0.330\times 0.371=0.123MPa\sqrt{m}$$

$$K_{0(LR=3)}=S.F\times K_{0(LR=1)}=1.588\times 0.371=0.589MPa\sqrt{m}$$

Considering the simple displacement between the statistical curves of the tested SCB group, a parameter m also needs to be considered. From Table 4, it can be seen that the difference between the calculated values of *m* at different loading rates is small and responds to the fact that the degree of material discretization has little effect on the displacement of the Weibull curve. The Weibull parameters of the curve can be obtained from *LR* = 1 mm/min and the Weibull probability parameters of asphalt concrete for *LR* = 0.05, 3 mm/min can be predicted as follows:

$$m_{\left(LR=1\right)}=m_{\left(LR=0.05\right)}=m_{\left(LR=3\right)}\approx 9$$

**Figure 6:**
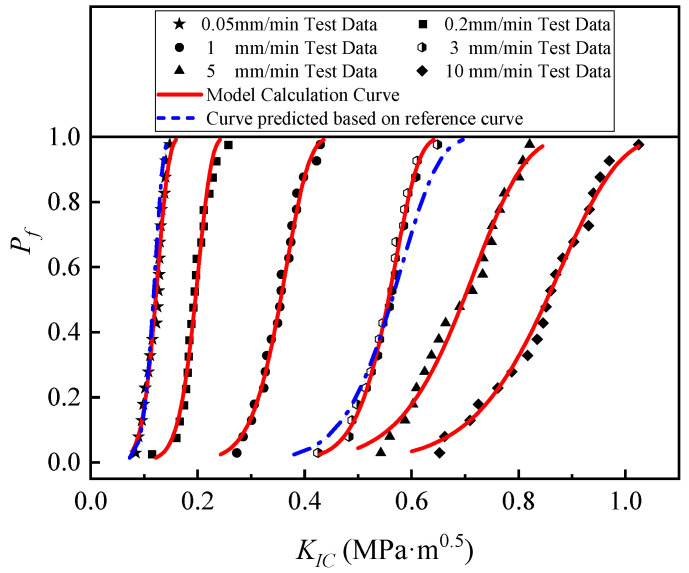
Comparison between *K_IC_* calculation curves and prediction curves of the two-parameter Weibull model under different loading rates.

The statistical probability curve of the tested SCB specimen at loading rates of 0.05 and 3 mm/min was obtained using the predicted Weibull parameter, as shown in Figure 6. Figure 6 compares the calculated and predicted two-parameter Weibull curves for different loading rates with the experimental data, which are statistical results obtained with reference to the experimental data for *LR* = 1 mm/min. As can be seen from the figure, the accuracy of the predicted hydraulic asphalt concrete fracture toughness values are all high, and the predicted curve specimen probability data are in good agreement with the test values. According to the reference Weibull parameter, the fracture toughness behaviors of the tested SCB specimens at different loading rates can be better estimated, which means that only 3–4 sets of statistical fracture toughness data need to be tested for a certain proportioning to predict the Weibull probability curves for an arbitrary desired loading rate without the need to conduct additional fracture toughness tests.

Table 5 compares the average *K_IC_* values with the *K*_0_ values for the predicted and test results for the SCB specimens with two different loading rates. It can be seen that the error values of the prediction models are low, and the error ranges are all less than 5%, indicating that the two-parameter Weibull distribution curves are all capable of predicting the effects of different loading rates on the Type I fracture toughness of hydraulic asphalt concrete using the SCB test.

The applicability of the Weibull statistical model in predicting the fracture toughness of asphalt concrete materials was also investigated by a previous scientific article [22], where the test results of the Type I fracture mode were used to predict the Type II fracture results of asphalt concrete, which were obtained under one loading rate condition, whereas in the present study, the Weibull model was used to predict the law of influence of the test results on the Type I fracture mode with different loading rates, and to compare the error ranges of the predicted average *K_IC_* and the model parameter *K*_0_ at different loading rates.

**Figure 7:**
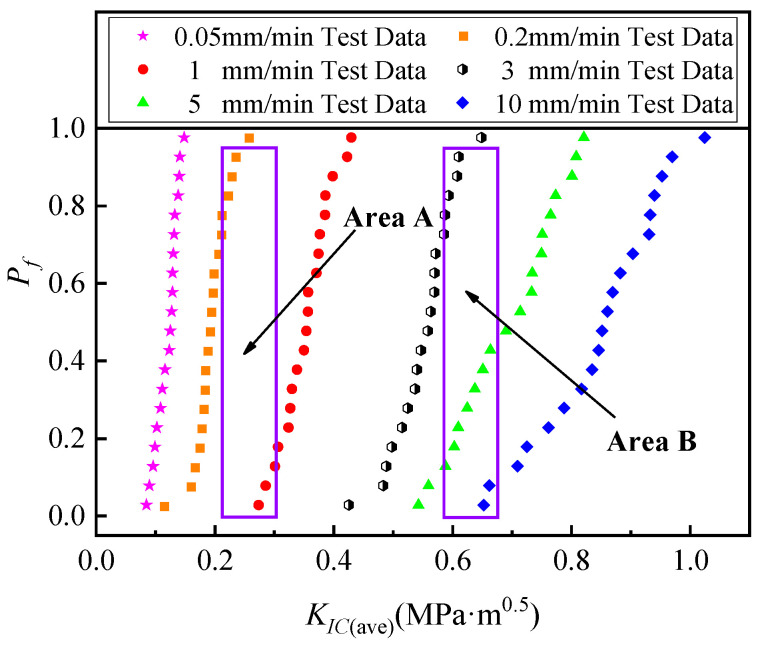
Considering the uncertainty of the effect of the loading rate on *K_IC_* for finite repetitions of the test.

Based on the basic principle of statistics, a more accurate analysis and estimation can be achieved by increasing the specimen capacity. A large number of repetitive SCB tests are conducted at different loading rates, and the results indicate that the loading rate has a significant effect on the cracking resistance of hydraulic asphalt concrete, which is a finding that may not be justified if the number of tests is too small. As the test data shown in Figure 7, “Area A” or “Area B” were obtained from fewer tests; it can be seen that the average *K_IC_* values under different loading rates are similar, which is the randomness of the specimen aggregate distribution leading to the uncertainty of the analysis. Therefore, by testing these 3–5 limited SCB specimens, it cannot be said with certainty that the loading rate has a significant effect on the fracture toughness of asphalt concrete.

According to the results of this study, by assuming a simple displacement factor, the fracture toughness distribution curve under any desired loading rate can be predicted based on the Weibull statistical curve of the reference test condition, and the appropriate Weibull distribution curve can be constructed by shifting the Weibull distribution curve given in this paper to the left or to the right using the displacement factor. From the constructed curves, it is possible to determine the range of variation between the upper and lower values of *K_IC_* at the desired loading rate.

Although the effect of the loading rate on the fracture resistance characteristics of asphalt concrete is considered in this paper, the Type I fracture toughness of different asphalt concretes varies with mix composition and environmental conditions, such as the test temperature and the maximum aggregate size, and different grades of asphalt also change the fracture toughness of asphalt concrete materials.

On the other hand, in semicircular bending (SCB) experiments, potential sources of error include the following: sample preparation errors: the sample preparation process may be affected by the operator’s skill level, the nature of the material, etc.; test equipment errors: there may be errors in the test equipment, e.g., inhomogeneous loading of the loading machinery; and environmental condition errors: variations in environmental conditions, such as temperature. Therefore, suggestions to reduce these errors include developing standardized operating procedures to ensure consistency in sample preparation and testing; regularly checking and calibrating the test equipment to ensure its accuracy and stability; conducting multiple repetitions of the test and calculating the average value to reduce the impact of random errors; and strictly controlling the temperature of the test environment.

## 4. Minimum Number of Specimens for Semicircular Bending Test *K_IC_*

### 4.1. Preliminary Analysis of K_IC_ Data

In order for the fracture toughness values obtained from the tests to better represent their overall mean values, it is necessary to provide a large specimen of data for the *K_IC_* test values of asphalt concrete, and the minimum number of specimens for the *K_IC_* of asphalt concrete was calculated using statistical methods. In practical engineering applications, for the distribution of the *K_IC_* for a set of data, the normal distribution or lognormal distribution are more often used [33], and for the distribution characteristics of the *K_IC_* in rock and concrete boundaries, the lognormal distribution or Weibull distribution are more often used [34]; therefore, the distribution of the *K_IC_* for hydraulic asphalt concrete is first explored here using a lognormal distribution for the time being.

When examining the characteristics of the *K_IC_* distribution, it is usually assumed that the *K_IC_* obeys a lognormal distribution. A random variable of *K_IC_* is denoted by X, for which its values are denoted by *x*; if X follows a lognormal distribution, X = lgX obeys a normal distribution, and there is the following equation:

(9) $$f(x)=\frac{1}{x\sigma_{1}\sqrt{2\pi }\times \ln10}EXP\left[-\frac{{\left(\mathrm{lg}x-\mu_{1}\right)}^{2}}{2\sigma_{1}^{2}}\right]$$

The Pearson *χ*^2^ test method requires that the specimen capacity must be greater than or equal to 50, and the specimen capacity of this test does not meet the requirements, so the normal probability coordinate paper test was used: if there is a larger specimen observation of *x_i_* (*i* = 1, 2, …, *n*), then the estimate *p_i_* corresponding to *x_i_* can be given by the following equation based on the average rank theory:

(10) $$p_{i}=1-\frac{i}{N+1}$$

where *i* is the number of tests (*i* = 1, 2, 3, …, *N*) ordered by the specimen data from smallest to largest, and *N* is the total number of tests at each loading rate.

**Figure 8:**
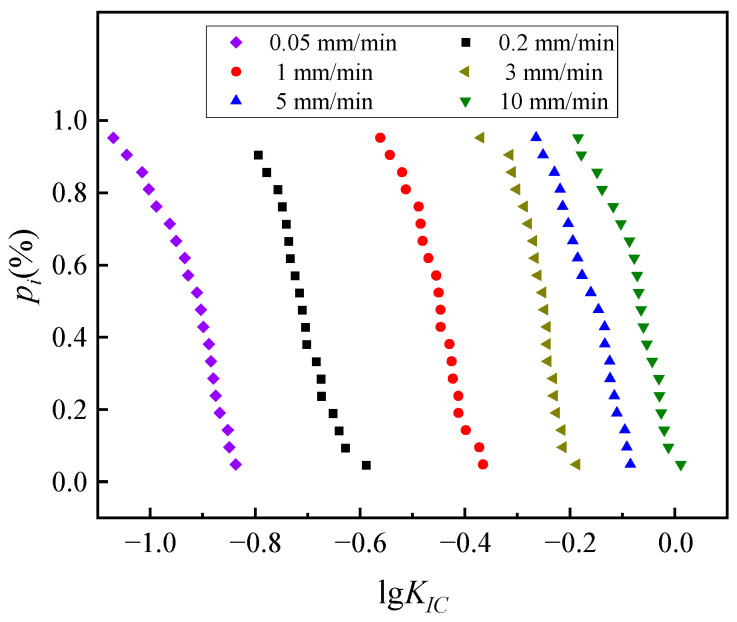
Normal probability coordinates (*x_i_*, *p_i_*).

**Figure 9:**
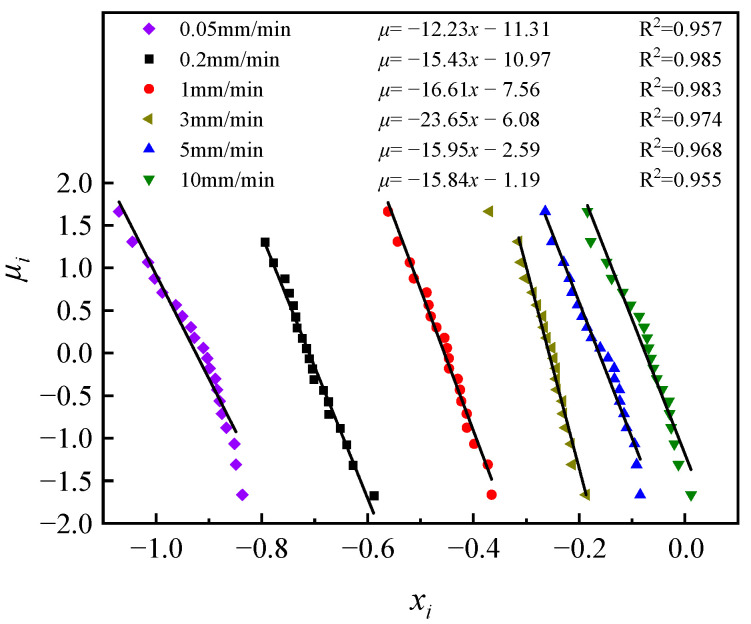
Normal probability coordinates (*x_i_*, *µ_i_*).

This tests whether the *K_IC_* obeys a lognormal distribution, that is, whether X = lg*K_IC_* obeys a normal distribution. However, this method lacks a quantitative criterion for whether *P_i_* − *x_i_* is linear or not; thus, the corresponding standard normal variable can be found from *P_i_*, taking the value of *µ_i_*, including the test data in Table 6. The table shows the data for the loading rate of 10 mm/min, and in converging (*x_i_*, *µ_i_*)(*i* = 1, 2, ..., 20) into normal probability coordinates, as shown in Figure 8, and a linear regression of (*x_i_*, *µ_i_*), as shown in Figure 9, the linear correlation factor *R*^2^ is greater than 0.95 for all six loading rates, so it can be assumed that the *K_IC_* basically obeys a lognormal distribution.

### 4.2. The Minimum Number of K_IC_ Specimens Is Determined

Statistically indicated, $x=\mathrm{lg}K_{IC}\sim N(\mu ,\sigma^{2})$. The mean value $\bar{x}$ and the standard deviation *s* of a specimen with a capacity of m can form a t-variable as follows [33]:

(11) $$t=\frac{\bar{x}-\mu }{s/\sqrt{m}}$$

with

(12) $$P\left\{\left|\frac{\bar{x}-\mu }{s/\sqrt{m}}\right|\leq t_{\left(\alpha /2\right)}(m-1)\right\}=1-\alpha =\gamma$$

where $\frac{\bar{x}-\mu }{s/\sqrt{m}}$ falls within the interval $t_{\left(\alpha /2\right)}(m-1)$ with confidence level *γ*; *μ* is the expected value, which can be calculated using the great likelihood estimation method; *α* is the significance level; and *m* is the specimen capacity. From this, we can derive the following:

(13) $$\left|\frac{\bar{x}-\mu }{\bar{x}}\right|\leq \frac{s}{\bar{x}}\frac{t_{\left(\alpha /2\right)}(m-1)}{\sqrt{m}}$$

If the relative deviation of the mean value $\bar{x}$ from *μ* at confidence level *γ* is required to be less than *δ*, the right-hand side of Equation (13) should be ≤*δ*:

(14) $$\frac{s}{\bar{x}}\leq \frac{\delta \sqrt{m}}{t_{\alpha /2}(m-1)}$$

where $t_{\alpha /2}(m-1)$ can be found in the t-distribution table [35]; however, at this point, *δ* is only the relative deviation of the mean value of $\mathrm{lg}K_{IC}$ (accuracy indicators). It is the relative deviation of *K_IC_* that needs to be controlled; if the corrected relative deviation δ* of *K_IC_* is given, then the following equation is used:

(15) $$\delta =\frac{\mathrm{lg}(1+\delta^{*})}{\bar{x}}$$

The minimum number of *K_IC_* specimens corresponding to the lognormal distribution is as follows:

(16) $$s\leq \frac{\ln(1+\delta^{*})\sqrt{m}}{t_{\alpha /2}(m-1)}$$

In this study, based on the *K_IC_* test data of hydraulic asphalt concrete, it was found that the standard deviation of the lognormal variable at a loading rate of 10 mm/min was maximized to $\bar{x}$ = 0.848, *s* = 0.054. This means that the maximum number of specimens is required for this condition, approximating the $s/\bar{x}$ value of the coefficient of variation for a small specimen with the $s/\bar{x}$ value of the coefficient of variation for a large specimen to estimate the minimum number of specimens. The confidence and accuracy indexes should be selected according to the actual requirements of the results [36]; typically, with *γ* = 0.9 to 0.95 and a relative deviation (accuracy index) *δ* = 0.05 to 0.10, this test takes *γ* = 0.95, δ* = 0.05.

When *m* = 7, $t_{0.025}(6)=2.4469$ and $s=0.054>\frac{\ln(1+0.05)\sqrt{7}}{2.4469}=0.052$: unsatisfied.
When *m* = 8, $t_{0.025}(7)=2.3646$ and $s=0.054\approx \frac{\ln(1+0.05)\sqrt{8}}{2.3646}\approx 0.058$: critical.
When *m* = 9, $t_{0.025}(8)=2.3060$ and $s=0.054<\frac{\ln(1+0.05)\sqrt{9}}{2.3060}=0.063$: satisfied.

It can be concluded that for the hydraulic asphalt concrete SCB test, the minimum number of specimens for *K_IC_* when *γ* = 0.95 and δ* = 0.05 corresponds to an estimated 9 specimens.

## 5. Conclusions and Discussion

Through the SCB test of hydraulic asphalt concrete, the following conclusions are drawn based on using the Weibull model to study the fracture index *K_IC_* of asphalt concrete under different loading rates:

- The loading rate has a significant effect on the critical stress intensity factor of hydraulic asphalt concrete, and the *K_IC_* dispersion is large at each loading rate, with the dispersion of the data at a loading rate of 10 mm/min differing by a factor of nearly 10 in the variance value compared to 0.2 mm/min. Consideration of the average fracture toughness values obtained from a limited number (3–5) of repetitive tests does not necessarily provide a suitable index for indicating actual damage behavior.
- The two-parameter Weibull model fits the distribution of the fracture strength of hydraulic asphalt concrete well and with high accuracy, thus allowing a better evaluation of the actual fracture behavior of the material; by assuming a simple displacement factor, distribution probability curves can be predicted for any desired loading rate based on the parameters of the referenced Weibull model.
- On the basis of existing tests, the *K_IC_* of hydraulic asphalt concrete was found to obey a lognormal distribution using statistical methods, and the minimum number of specimens for the SCB test was estimated to be 9 when *γ* = 0.95 and under the condition that the relative deviation does not exceed 5%.

Although the effect of the loading rate on the fracture resistance characteristics of asphalt concrete is considered in this paper, the Type I fracture toughness of asphalt concretes varies with mix composition and environmental conditions, such as the test temperature and the maximum aggregate size, and different grades of asphalt also change the fracture toughness of asphalt concrete materials. In order to obtain a deeper understanding of the fracture characteristics of asphalt mixtures, the dependence of each parameter on the fracture properties should be systematically considered, and the cracks in asphalt concrete should be deeply investigated using unequal spacing and diagonal cuts.

On the other hand, potential sources of error in semicircular bending (SCB) experiments include the following: the sample preparation process may be affected by factors such as the skill level of the operator and the nature of the material; the test equipment may contain errors, such as uneven loading by the loading machinery; and variations in the environmental conditions, such as temperature. Therefore, in order to reduce these errors, it is recommended that standardized operating procedures be established to ensure consistency in sample preparation and testing; test equipment be checked and calibrated regularly to ensure its accuracy and stability; multiple repetitions of the test be carried out and averages calculated in order to reduce the effect of random errors; and the temperature of the test environment be strictly controlled.

## Figures and Tables

**Table 1:** Asphalt performance index.

Test Item	Units	SL 501-2010	Specimen Test Results
Needle penetration (25 °C,100 g, 5 s)	0.1 mm	60~80	66.5
Ductility (5 cm/min, 10 °C)	cm	≥20	55
Ductility (5 cm/min, 15 °C)	cm	-	>150
Softening point (Globe method)	°C	≥45	53.0
Flash point	°C	≥260	320
Mass change after heating in a film oven	%	-	−0.06
Residual needle entry ratio (25 °C)	%	≥61	83.3
Residual ductility (5 cm/min, 10 °C)	cm	≥6	15.1

**Table 2:** Mineral mixture ratio.

Raw Material	Grain Size/mm							Pitch
	19–16	16–13.2	13.2–9.5	9.5–4.75	4.75–2.36	2.36–0.075	<0.075	
**Ratio%**	6.6	6.8	10.5	18.2	15.9	30.0	12.0	7.5

**Table 3:** Results of statistical analysis on the data at different loading rates.

Fracture Indicator	Loading Rate (mm/min)	*K_IC_* Mean Value (Mpa·m^0.5^)	*K_IC_* Maximum	*K_IC_* Minimum	*K_IC_* Spread	Median *K_IC_*	Variance Results
*K_IC_*	0.2	0.1945	0.2578	0.1149	0.1429	0.1934	0.0010
	1	0.3533	0.4309	0.2745	0.1564	0.3562	0.0017
	5	0.6929	0.8232	0.5442	0.2790	0.7038	0.0070
	10	0.8479	1.0271	0.6540	0.3731	0.8584	0.0102

**Table 4:** Parameters of the two-parameter Weibull curve of SCB specimens at different loading rates.

Loading Rate	*K_0_*	m	*K* Mean	*R* ^2^
(mm/min)	(MPa·m^0.5^)		(MPa·m^0.5^)	
0.2	0.205	9.004	0.1945	0.9613
1	0.371	9.324	0.3533	0.9847
5	0.731	8.509	0.6929	0.9754
10	0.892	8.886	0.8479	0.9870

**Table 5:** Comparison between the calculated and predicted values of the two-parameter Weibull model test data of SCB specimens.

Model Parameters	*LR* = 0.05 mm/min			*LR* = 3 mm/min		
	Test Data	Predicted Values	Error	Test Data	Predicted Values	Error
Average *K_IC_*	0.121	0.116	4.13%	0.553	0.561	1.45%
*K*_0_	0.129	0.123	4.65%	0.574	0.589	2.61%

**Table 6:** *K_IC_* data and processing of hydraulic asphalt concrete (10 mm/min).

i	*K_IC_*/MPa·m^0.5^	lg*K_IC_*	*p_i_*	*µ_i_*
1	0.6540	−0.1844	0.952	1.6646
2	0.6637	−0.1780	0.905	1.3106
3	0.7113	−0.1480	0.857	1.0669
4	0.7271	−0.1384	0.810	0.8779
5	0.7633	−0.1173	0.762	0.7128
6	0.7897	−0.1025	0.714	0.5651
7	0.8193	−0.0865	0.667	0.4316
8	0.8371	−0.0772	0.619	0.3029
9	0.8481	−0.0715	0.571	0.1789
10	0.8539	−0.0686	0.524	0.0602
11	0.8629	−0.0640	0.476	−0.0602
12	0.8716	−0.0597	0.429	−0.1789
13	0.8848	−0.0531	0.381	−0.3029
14	0.9056	−0.0430	0.333	−0.4316
15	0.9331	−0.0301	0.286	−0.5651
16	0.9350	−0.0292	0.238	−0.7128
17	0.9424	−0.0257	0.190	−0.8779
18	0.9551	−0.0199	0.143	−1.0669
19	0.9723	−0.0122	0.095	−1.3106
20	1.0271	0.0116	0.048	−1.6646

## Data Availability

Data are contained within the article.

## Notation

*K_IC_*	critical stress intensity factor
*P*	critical load
*r*	radius
*t*	thickness
*a*	length of the cutout
*Y_I_*_(0.8)_	Type I fracture stress intensity factor
*P_f_*	probability of failure
*K_0_*	normalization factor
*LR*	loading rate
*S.F*	shift factor
*m*	size of deviation
$\bar{x}$	mean value
*s*	standard deviation
*μ*	expected value
*m*	sample size
*γ*	confidence level
*α*	significance level
*δ*	relative deviation
*δ**	corrected relative deviation
